# Insight on cytotoxic NHC gold(I) halide complexes evaluated in multifaceted culture systems

**DOI:** 10.1016/j.crtox.2024.100174

**Published:** 2024-05-23

**Authors:** Vincenza De Gregorio, Alessandra La Pietra, Andrea Candela, Carlo Oliviero, Ida Ferrandino, Diego Tesauro

**Affiliations:** a Department of Biology University of Naples “Federico II”, Via Cinthia 80126, Napoli, Italy; b Department of Experimental Medicine, Section of Biotechnology, Medical Histology and Molecular Biology, University of Campania “Luigi Vanvitelli”, 80138 Naples, Italy; c Department of Pharmacy and Interuniversity Research Centre on Bioactive Peptides (CIRPeB), University of Naples “Federico II”, Via Montesano 49, 80131 Naples, Italy

**Keywords:** Embryotoxicity, NHC-Au complexes, Zebrafish model, Bovine model, 2D culture system, Apoptosis

## Abstract

Gold complexes can be a useful system in the fight against cancer. Although many studies have been carried out on in vitro 2D cell culture models embryotoxic assays are particularly lacking. Embryotoxicity and DNA damage are critical concerns in drug development. In this study, the effects of a new N-Heterocyclic carbene (NHC)-Au compound (Bromo[1,3-di-4-methoxybenzyl-4,5-bis(4-methoxyphenyl)imidazol-2-ylidene]gold(I)) at different concentrations were explored using multifaceted approach, encompassing 2D cancer cell cultures, *in vivo* zebrafish and in vitro bovine models, and compared with a consolidated similar complex (Bromo[1,3-diethyl-4,5-bis(4-methoxyphenyl)imidazol-2-ylidene]gold(I)). The results obtained from 2D cancer cell cultures revealed concentration-dependent effects of the gold compounds by estimating the cytotoxicity with MTT assay and cellular damage as indicated by LDH release. Selected concentrations of gold complexes demonstrated no adverse effects on zebrafish embryo development. However, in bovine embryos, these same concentrations led to significant impairments in the early developmental stages, triggering cell apoptosis and reducing blastocyst competence. These findings underscore the importance of evaluating drug effects across different model systems to comprehensively assess their safety and potential impact on embryonic development.

## Highlights

• N-Heterocyclic carbene (NHC)-Au compounds show promising cytotoxicity in cancer cells.
• Evaluation across zebrafish and bovine embryos reveals diverse effects, emphasizing varied model assessments.
• Study deepens embryonic toxicity understanding, guiding safer gold compound applications in cancer treatment.

## Graphical abstract

**Figure:**
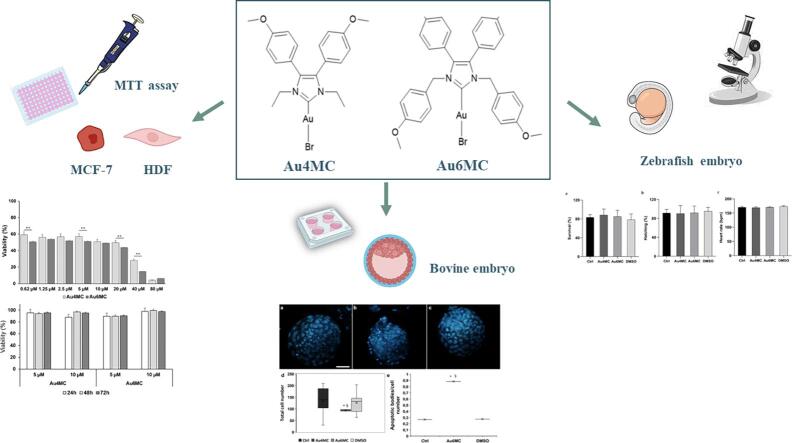

## 1 Introduction

Cisplatin (CDDP) was approved by the U.S. Food and Drug Administration (FDA) in 1978 for the treatment of genitourinary tumors, but severe side-effects and resistance can occur during the treatment process (Kelland, 2007). Therefore, scientists have been involved in the search for new transition-metal complexes that can perform as antiproliferative agents towards tumor cells (Clarke et al., 1999). Gold complexes can exert their strong growth inhibitory effects with mechanisms of action not similar to cisplatin, exhibiting non-cisplatin-like mechanisms of action, showing unalike pharmacodynamic and pharmacokinetic properties (Ott, 2009, Komeda and Casini, 2012). The first gold complex approved by FDA in 1985 was auranofin (AF), initially approved as an anti-arthritis agent but it has been studied for potential therapeutic application in a number of other diseases including cancer (Roder and Thomson, 2015). A promising class of bi-coordinate Au (I) complexes bears N-Heterocyclic carbene (NHC) as ligands that give rise to strong carbon-to-metal bonds, resulting in highly stable complexes in physiological conditions (Porchia et al., 2018). Several studies were carried out on NHC-Au complexes to find available targets and to investigate their behavior inside cancer cells (Rubbiani et al., 2014, Bazzicalupi et al., 2016, Augello et al., 2023). Very recently, Lu et al. demonstrated *in vivo* xenograft study that NHC gold complex dual targeting ER and TrxR, had excellent antiproliferative activity toward MCF-7 cells in mice mode (Lu et al., 2023). However, most of the toxicological high throughput studies have been focused on in vitro 2D cell culture models which cannot denote the safety assessment. Alternative approaches in reproductive toxicity include computational methods (Liu et al., 2022) and studies on embryonic stem cells (Tandon and Jyoti, 2012), highlight the diverse strategies employed in this field. Due to their metabolic and cell cycle resemblances to cancer cells, the reproductive cells, including embryos, provide a valuable model for investigating chemotherapeutical toxicity. Hence, the need to use more complex model systems as reliable tools that allow us a comprehensive understanding of the potential adverse effects associated with various compounds during toxicity assessments, with the ultimate aim of translating the findings into human applications (Erhirhie et al., 2018). The use of chemotherapeutics during pregnancy, breeding, or at a young age could pose a risk, so, it is important to test their toxicity *in vivo* during these stages (Gao et al., 2017). Zebrafish, a vertebrate approximately 71 % genetically identical to humans (Howe et al., 2013), is an excellent model organism that lends itself well to the study of the toxicity of new compounds or pollutants in both medical (Hung et al., 2019, Van Sebille et al., 2019) and ecotoxicological fields (Capriello et al., 2019, Capriello et al., 2021, La Pietra et al., 2024). Its embryos are used for embryotoxicity testing because of rapid reproduction, transparency, and rapid development (Yang et al., 2009). Several studies to evaluate the activity of NHC complexes of gold, iron or ruthenium have used zebrafish embryos for *in vivo* testing (Oehninger et al., 2013, Farooq et al., 2015, Lenis-Rojas et al., 2021). On the other hand, bovine in vitro fertilization (IVF) serves as a robust platform for studying the toxicity of chemotherapeutic agents due to their similarity to human reproductive physiology. This model provides a controlled environment that allows for comprehensive investigations into the effects of chemotherapeutic compounds on crucial reproductive events, such as oocyte maturation, fertilization, and subsequent the early embryonic development (Santos et al., 2014, Asimaki et al., 2022). In the current study we selected two NHC Au complexes Bromo[1,3-diethyl-4,5-bis(4-methoxyphenyl)imidazol-2-ylidene]gold(I) (Au4MC) and Bromo[1,3-di-4-methoxybenzyl-4,5-bis(4-methoxyphenyl)imidazol-2-ylidene]gold(I) (Au6MC) and evaluated their cytotoxic activity on Human breast cancer (MCF7) and normal Human dermal fibroblast (HDF) cells. To improve knowledge on toxicity and bioactivity of NHC gold complexes we reported *in vivo* test in zebrafish and in vitro culture of bovine embryos. This comparative approach enhances our understanding of the potential impact of chemotherapeutic agents on humans and supports the development of safer and more targeted therapeutic strategies.

## 2 Materials and methods

Solvent and chemical reagents were purchased and used as received without further purification (Sigma-Aldrich, Steinheim, Germany), ^1^H NMR spectra were acquired with Brucker 400 MHz, ESI mass spectra were recorded in positive mode with an Applied Biosystems mass spectrometer equipped with a triple quadrupole mass analyzer. Compounds 4,5-bis(4-methoxyphenyl)imidazole, 1,3-diethyl-4,5-bis(4-methoxyphenyl)imidazole bromide, and Bromo[1,3-diethyl-4,5-bis(4-methoxyphenyl)imidazolin-2-ylidene]gold(I) were prepared as described in literature (Liu et al., 2011a, Liu et al., 2011b).

### 2.1 Chemistry synthesis

#### 2.1.1 Synthesis of 1,3-dimethoxybenzyl-4,5-bis(4-methoxyphenyl)imidazole chloride (1)

To a solution of 4,5-bis(4-methoxyphenyl)imidazole (1.00 mmol) in 5.00 mL of CH_3_CN 1.50 mmol of K_2_CO_3_ were added and stirred for 15 min. Subsequently 2.20 mmol of 4-Methoxybenzyl chloride were added dropwise and heated to reflux for 3 h. Additional 2.0 mol were added and the mixture was stirred for 48 h. The solvent was removed in vacuo and the residue was dissolved in 20.0 mL of ethyl acetate and washed twice with 15.0 mL of brine. The organic phase was dried over anhydrous Na_2_SO_4_. Then removal of solvent under reduced pressure gave the crude product, which was purified by flash chromatography (98:2 CH_2_Cl_2_:CH_3_OH) achieving a pale yellow glassy solid. (174 mg, yields 35 %) ESI + -MS, *m*/*z*: 499 [M] + .

#### 2.1.2 Synthesis of 1,3-dimethoxybenzyl-4,5-bis(4-methoxyphenyl)imidazole hexafluorophosphate.

To a solution of 1,3-dimethoxybenzyl-4,5-bis(4-methoxyphenyl)imidazole chloride (0.187 mmol) in 10.0 mL of acetone 0.281 mmol of KPF_6_ were added and stirred for 20 h at room temperature. The solution was filtered over a bed of celite and dried under vacuum. The residue was wash with little portion of diethyl ether achieving the product as pale yellow solid (109 mg yields 90 %) ESI + -MS, *m*/*z*: 499 [M]+.

#### 2.1.3 Synthesis of 1,3-dimethoxybenzyl-4,5-bis(4-methoxyphenyl)imidazole gold (I)

To a stirred solution of 1,3-dimethoxybenzyl-4,5-bis(4-methoxyphenyl)imidazole Hexafluorophosphate (0.17 mmol) in 35 mL of dichloromethane/methanol (6:1) was added silver(I) oxide (24 mg, 0.101 mmol) under N_2_, and the reaction mixture was allowed to stir for 12 h in the dark. Me_2_SAuCl (24.5 mg, 0.085 mmol) and LiBr (147 mg 1.70 mmol) were added, and the suspension was stirred for an additional 5 h. The suspension was filtered over a bed of Celite. The filtrate was concentrated under vacuum and was purified on column chromatography on silica gel (Ethyl Acetate/Hexane 7:3) achieving a glassy solid (40 mg yields 60 % ESI + -MS, *m*/*z*: 776 [M] + ).

### 2.2 Cell culture

Human breast cancer cells (MCF7) were obtained from American Type Cell Culture (ATCC, Manassas, VA, USA) and grown in Roswell Park Memorial Institute Medium (RPMI 1640, BioWest, Nuaillé − France) with the addition of 10 % fetal bovine serum (FBS, Sigma-Aldrich, Milan − Italy), 100 μg mL^−1^ of L-glutamine, and 100 IU mL^−1^ of streptomycin/penicillin (Pen/Strep; BioWest, Nuaillé − France) at 37 °C with 5 % CO_2_. Human dermal fibroblasts (HDF) were grown in Dulbecco’s Modified Eagle’s Medium (DMEM, Gibco, Dublin, Ireland) supplemented with 10 % FBS, 100 μg mL^−1^ of L-glutamine, and 100 IU mL^−1^ of streptomycin/penicillin at 37 °C with 5 % CO_2_. After reaching 80 % confluence, the cells were harvested with trypsin/EDTA (BioWest, Nuaillé − France), centrifuged at 220 x *g* for 5 min and seeded in 11 cm-petri dish.

#### 2.2.1 Cytotoxicity evaluation (MTT assay)

The 3-(4,5-dimethylthiazol-2-yl)-2,5-diphenyltetrazolium (MTT) assay was carried out on the MCF7 and HDF cell lines to determine the selective activity of anticancer Au compounds. Firstly, the Au compounds, produced as described at item 2.1., were dissolved in dimethyl sulfoxide (DMSO, Sigma-Aldrich, Milan − Italy) to produce a stock solution and then, two-fold serial dilutions were prepared (from 0.62 to 160 μM). To assess cell viability, the cells were seeded in 96-well micro-plates and starved in 2 % FBS-media 24 h before the treatments. MTT tests were performed with an exposure for 24, 48 and 72 h according to the manufacturer’s instructions (Sigma Aldrich, Milan − Italy). Briefly, MTT (500 μg/mL – final concentration) was added to each well and the plates were incubated further for 1–4 h and washed three times. The supernatant was removed and DMSO was added to each well to solubilize the formazan crystals. The optical density of the samples in each well was measured by reading at 570 nm with a microplate spectrophotometer (Synergy H4 plate reader, Gene5 2.07 software). All the analyses were carried out in triplicate. The maximal inhibitory concentration (IC50) values on MCF7 cells for Au4MC (5.53 μM) and Au6MC (5.03 μM) were estimated using linear regression (Table 1).

**Table 1:** The IC50 values for Au4MC and Au6MC.

Compound	IC50
Au4MC	5.53 μM
Au6MC	5.03 μM

#### 2.2.2 LDH analysis on spent media

The cell damage was characterized on spent media by using LDH assay (Sigma-Aldrich, Milano, Italy) on MCF7 cell line. Spent culture medium from cell/embryo was collected, centrifuged at 200g for 10 min to remove cell debris, and 50 μL of supernatant was added to each well of 96-well plates together with positive controls (DMSO 50 %) or NADH standards at different concentrations. After 3 min, the absorbance at 450 nm was measured with a microplate reader (Synergy HTX Multi-Mode Reader, Agilent, Milano, Italy) at the initial test time, Ti. Subsequent measurements were conducted at 5-min intervals. The last measurement recorded was the penultimate measurement, or the value before the most active sample reaches or exceeds the end of the linear range (Tf). LDH activity at each time point was evaluated according to the following equation:

(1) LDH=Bxdilutionfactor/ReactiontimexV

where B is the amount (nmole) of NADH generated between Ti and Tf, the reaction time indicates the difference between Tf and i, and V is the sample volume. LDH was expressed as nmole/min/mL (that is mUnit/mL). Measurements were performed in duplicate.

### 2.3 Zebrafish model

#### 2.3.1 Zebrafish breeding

Adult zebrafish were housed in oxygenated tanks at the Department of Biology of the University of Naples Federico II with a 14 h:10 h light/dark photoperiod, at a temperature of 28.0 °C, pH of 7.5. Zebrafish were fed with a commercial diet (TetraMin Tropical Flake Fish®) supplemented with live Artemia sp. nauplii (Westerfield, 2000). All experiments were conducted in accordance with the guidelines and policies dictated by European regulations on the wellness of animals employed for experimental purposes (Directive 2010/63/EU) and the Italian animal protection standards (D.lgs. 26/2014).

#### 2.3.2 Embryos treatment

Eggs were collected by siphoning, selected under a stereomicroscope (Leica Zoom 2000), and transferred into E3 medium (5 mM NaCl, 0.17 mM KCl, 0.33 mM CaCl_2_⋅2H_2_O, 0.33 mM MgSO_4_) at 28 °C.

At 6 h post fertilization (hpf), four groups with ten embryos each one, in duplicate (20 embryos for group in total), were set up in 6-well microplates in according to La Pietra et al., (2024); three groups were treated with 10 mL of Au4Mc 5.53 μM, Au6Mc 5.03 μM or DMSO 0.01 %, respectively, and one group was exposed to only E3 medium as control group. Embryos treated were incubated under static conditions and monitored up to 72 h (h) of treatment.

Experiments were performed in triplicate.

#### 2.3.3 Toxicity parameters

The survival rate was evaluated at 24 h, 48 h and 72 h of treatment, and the hatching rate at 48 h of treatment. Both parameters were calculated by the ratio between the number of dead embryos, or hatched larvae, and the total number of embryos (or larvae). The heart rate was measured at 72 h of treatment in six individuals for each group (Wang et al., 2022), for a 15 s period, and then calculated per minute (Monaco et al., 2017). Each larva was placed in a hanging drop slide and observed under a light microscope. The data are the average of three measurements per larvae.

### 2.4 Bovine embryo culture

#### 2.4.1 Oocyte retrieval and in vitro maturation

Bovine ovaries were collected from Di Tella S.R.L. slaughterhouse (Slaughterhouse Straccione, San Marcellino, Caserta, Italy; CEE accreditation number 1403/M) and transported to the laboratory approximately 3 h at 33 °C. Then, the bovine ovaries were washed in pre-warmed 0.9 % NaCl solution supplemented with 1 % Pen/Strep. Cumulus oocyte complexes (COCs) were collected by aspiration of individual follicles with an 18-gauge needle in 15 ml conical-bottom tubes (Falcon; Milan, Italy) containing 70 μl heparin (10 mg/mL) for approximately 10 ml of follicular fluid. COCs were, then, washed in wash medium (Stroebechmedia, Denmark) and matured in Nunc 4-well dishes for IVF (Termo Fischer Scientific, Roskilde, Denmark) containing 500 µl of IVM medium (Stroebechmedia, Denmark) for 22–24 h at 38.8 °C, in an incubator at 6 % CO_2_ in humidity atmosphere.

#### 2.4.2 Semen preparation

Frozen bovine semen of three bulls (0.5 ml straws; approximately 10x10^6^ spermatozoa per straw; motility after thawing > 70 %), was obtained from Intermizoo S.p.a (Padova, Italy). Straws were thawed in a water bath at 37.5 °C for 30 s and rinsed in 10 mL of semen wash medium (Stroebechmedia, Denmark) by centrifugation at 170 × g for 10 min. Then, sperm pellet was resuspended in 1 ml of semen wash medium, and the recovered spermatozoa were assessed for concentration and motility by using a Makler chamber placed on a microscope stage heated to 38.8 °C and were analyzed with a Nikon TE 2000 (Tokio, Japan) inverted microscope connected to a Basler Vision Technology A312 FC camera (Ahrensburg, Germany) with a positive phase contrast 10x objective. At least 400 cells and four/five fields were acquired and analyzed for each semen sample. Progressive motility and kinetics were evaluated with a Sperm Class Analyzer (SCA Microptic S.L., Barcelona, Spain) in terms of curvilinear velocity (VCL), straight-line velocity (VSL), and average path velocity (VAP). The following software settings were used: 25 frames/s, 10 frames/object, 10 μm/s velocity limit for slow spermatozoa, 25 μm/s velocity limit for average spermatozoa, 50 μm/s velocity limit for rapid spermatozoa, 50 % minimal linearity, and 70 % straightness for progressive fast spermatozoa.

#### 2.4.3 In vitro fertilization, embryos culture and treatments

For in vitro fertilization, 45–50 matured COCs for each well were suspended in 250 μl IVF Medium (Stroebechmedia, Denmark), inseminated with 250 μl of sperm suspension (2x10^6^/ml – motile sperm concentration). After 19–22 h of co-incubation at 38.8 °C and 6 % CO_2_, the COCs were transferred in IVC medium (Stroebechmedia, Denmark) and cumulus cells were removed by vortexing (1 mL volume, medium velocity for 2 min). Presumptive zygotes were collected, washed and divided into 4 groups (50 zygotes for each group): untreated group (CTRL), group treated with the concentrations chose based on IC50 from MTT assay: 5.03 μM of Au6MC or 5.53 μM of Au4MC, and group treated with DMSO 0.0168 %, which corresponds to the final concentration of DMSO used to prepare a solution containing 5.53 μM of Au4MC compound, estimating the potential cytotoxic effects of DMSO in the absence of the compound. Zygotes for each condition were incubated in groups of 50 (n = 2) in 500 μl of IVC medium (Stroebechmedia, Denmark) for 8 days at 38.8 °C, in a 6 % CO_2_, 6 % O_2_ and 88 % N_2_ gaseous atmosphere. Cleavage, 2–4 and 8-cell embryo rates were determined at day 3 post insemination (d3 p.i.). Blastocyst rates (blastocysts/cleaved embryos) were determined on day 8p.i. At least 100 zygotes were used for each culture condition. The apoptotic cells of blocked embryo were evaluated by determining the total fluorescence intensity after Hoechst 33,342 staining as described at 2.4.4. paragraph. Furthermore, the blastocysts obtained were fixed and labelled with Hoechst 33,342 to assess the blastocyst’s total cell number as well as the intact nuclei and apoptotic bodies ratio.

#### 2.4.4 Evaluation of embryo developmental stage after Au compounds treatments

To evaluate the potential of the compounds to interfere with embryo development, stereomicroscopy observation (phase-contrast inverted microscope, Leica-MZ6) and image acquisition using Motic Images Plus 3.0 software on 3d p.i. and 8d p.i. embryos were performed. The percentage of fertilization and the percentage of embryos at the stages of 2–4 cells, 8 cells and beyond (16 cells, compacted morulae, blastocysts) were, then, calculated for each condition as follow:

(2) $$\mathrm{Fertilization}\;rate\left(\%Fertilized\right)=\left(\mathrm{Fertilized}\;embryos/Total\;embryos\right)x100$$

and

(3) %2-4or8-cell/cleavedembryosx100

All data were calculated as cumulative percentages from two independent experiments. This study involved the use of approximately 500 embryos.

At the end of embryo culture, the samples were fixed and processed for staining to assess embryo quality (evaluation of cell number and apoptosis) after Au compound treatments. Briefly, on 8d p.i. bovine embryos that reached the blastocyst stage were washed and fixed in a paraformaldehyde solution (4 % PFA, 1 % FBS) at RT overnight. Then, blastocysts were washed three times (10 min for each) in a drop 1X PBS solution (1.5 mM KH_2_PO_4_, 154 mM NaCl, 2.7 mM NaH_2_PO_4_ in H_2_O milliQ) containing polyvinyl alcohol (PVA, 3 mg/ml) at RT and treated with a permeabilization solution (0.1 % Triton X-100 in 0.1 % sodium citrate) at 4 °C for 30 min. Subsequently, three 10-minute washes were carried out using 1X PBS + PVA solution. Then, the embryos were stained in 50 μl drops of 10 μg/mL Hoechst 33,342 solution (1:100 in 1X PBS-PVA solution) for 7 min at RT, washed three times in 1X PBS-PVA solution and mounted on a microscope slide closed with 24 x 24 mm coverslips. The observation was carried out using an epifluorescence microscope (Nikon Eclipse TE2000-U) connected to a Nikon DS-5Mc video camera. Samples were viewed at 40X (for embryo stage and cell counting) or 100X (for apoptosis) magnifications using the UV filter for Hoechst (λ_ex_ 100–400 nm, λ_em_ 460–490 nm). Micrographs were captured using the NIS-element software. Subsequently, image analysis was conducted as reported below.

#### 2.4.5 Cell count

To calculate the total cell number in bovine blastocysts, image analysis on Hoechst-stained samples using the Image J software we conducted. The 'total cell counter' tool was used, allowing us to count all cells within the blastocysts, including both the inner cell mass (ICM) and trophoectoderm cells.

#### 2.4.6 Evaluation of apoptosis on blocked embryos

To assess apoptosis in bovine blocked embryos under different culture conditions (CTRL, Au4MC, Au6MC, and DMSO), the Hoechst-labelled specimens were examined under a fluorescence microscope at 100X magnification. Apoptotic cells exhibit condensed nuclei rich in heterochromatin with intense fluorescence, while healthy cells display regular nuclei with scattered patches of heterochromatin. Image J software was used to quantify the total fluorescence intensity on binary images. Single cell was selected by using the ‘drawing or selection’ tools and the area was measured. Afterward, a background subtraction was conducted by selecting a small area surrounding the analyzed cell (w/o fluorescence). The following formula was applied to measure the total cell fluorescence intensity expressed as correlated total cell fluorescence (CTCF) by the formula:

(4) $$\mathrm{CTCF}=Integrated\;Density-\left(\mathrm{Area}\;of\;the\;selected\;cell\times Mean\;fluorescence\;of\;the\;background\right)$$

#### 2.4.7 Assessment of apoptotic bodies and intact cells in blastocysts

To quantify the count of intact cells and apoptotic bodies in blastocysts produced under different culture conditions (CTRL, Au6MC, and DMSO), a manual image analysis using the Image J software was performed following the method described in the literature (Filipova et al., 2018) with slight adjustments. No blastocysts were generated when Au4MC supplementation was utilized, leading to the exclusion of this sample from the analysis. The apoptotic bodies were discriminated on size and brightness intensity. A background subtraction, a conversion into a binary format (black − 0 and white − 1) and then, a manual counting was performed for intact nuclei. In contrast, an automatic counting for apoptotic bodies was performed by converting the image into a binary format using the ‘thresholding’ tool and the Watershed algorithm was employed to distinguish overlapping or closely adjacent cells. The final object count was conducted using the ‘Analyze particles’ tool with the following parameters for apoptotic bodies: size range of 0–250 pixels and circularity range of 0–––1. The total number of detected objects was exported to Microsoft Excel®. Subsequently, the analysis of the total count of intact nuclei and apoptotic bodies in the different samples was reported as ratio.

### 2.5 Statistical analysis

The experiments were carried out in triplicates, with each condition consisting of both technical duplicates and triplicates, and the data were expressed as mean ± SD. Statistical analysis of toxicity parameters data on 2D cell cultures, zebrafish and bovine embryos was performed using GraphPad Prism Software (version 8.02 for Windows, GraphPad Software, La Jolla, CA, USA). One-way analysis of variance (ANOVA) method followed by Tukey’s pairwise comparison tests were used for data analyses. Further, analysis of bovine embryo stages and total cell number was reported as cumulative percentages by using Fisher’s exact test for pairwise comparisons when an overall significance was detected. p-values < 0.05 were considered to indicate statistical significance.

## 3 Results

### 3.1 Selection and synthesis of gold complexes

**Fig. 1:**
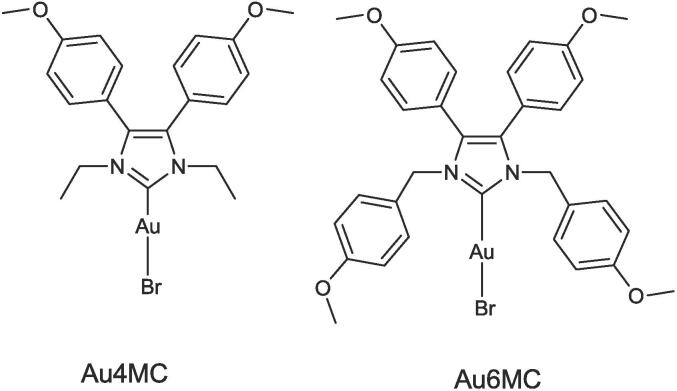
Chemical structures of the selected complexes Au4MC and Au6MC.

We selected one gold complex from the literature: Bromo[1,3-diethyl-4,5-bis(4-methoxyphenyl) imidazol-2-ylidene]gold(I) (Au4MC) (Fig. 1 left) (Liu et al., 2011a). The diaryl-substituted carbene as ligand is suitable to his high lipophilicity to cross cell and mitochondrial membranes. Moreover, this complex has been demonstrated to be active against MCF-7 cell line. The second one (Au6MC) was designed by replacing the ethyl groups with 4-methoxyphenyl moieties (Fig. 1 right). This complex retains the same lipophilicity and introduces a steric hindrance that can influence gold ion release. The gold ion is a cytotoxic agent by altering the metabolic mechanism of tumoral cells.

**Fig. 2:**
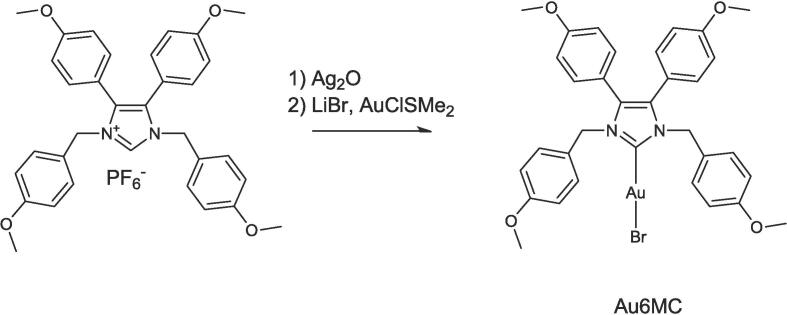
Scheme of synthesis of Au6MC.

Au6MC was obtained in one pot (Fig. 2) adding to two equivalents of the proligand in dichloromethane/methanol (6:1) solution, silver oxide, lithium bromide and chloride dimethyl sulfide gold complex. After that, the complex was purified by silica gel chromatography and later crystallization, it was identified by ESI MS experiments.

### 3.2 Assessment of cytotoxicity and cell damage on 2D cell culture models

**Fig. 3:**
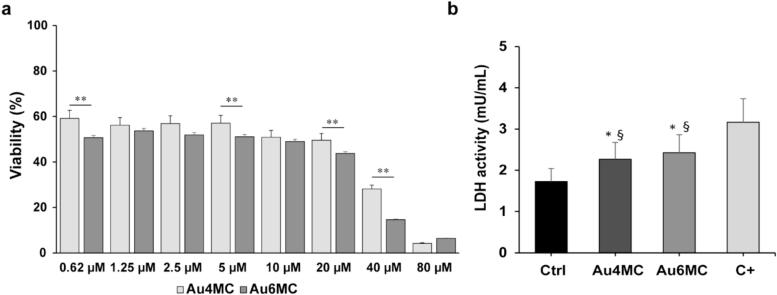
**Effects of Au4MC or Au6MC treatment on cell viability and LDH release on MCF7 cell line.** a) MCF7 were treated with different concentrations of Au4MC or Au6MC for 72 h. Cell viability was determined by using an MTT assay. Data are expressed as the percentage of control cells and are the means ± SD of three independent experiments, each performed in triplicate; ** p < 0.01. b) LDH activity (mUnits/mL) of MCF7-untreated sample (Ctrl), MCF7-treated samples (Au4MC and Au6MC), and MCF7-treated with high dose of DMSO (C + ). All the experiments were performed in triplicate, data are presented as means ± SD; * p < 0.05 *vs* Ctrl, § p < 0.05 *vs* C + .

To explore the ability of Au4MC and Au6MC compounds to inhibit the growth of mammalian cancer cells (MCF7) and healthy cells (HDF) at various concentrations (0.62, 1.25, 2.5, 5, 10, 20, 40, 80, 160 μM), MTT cytotoxicity assay was performed at 72 h post incubation. The graph (Fig. 3a) indicated that all examined concentrations of the compounds led to a decrease in cell viability at 72 h post-treatment in a dose-dependent manner. The IC50 values for both Au compounds, calculated using regression lines were 5.53 µM for Au4MC and 5.03 µM for Au6MC (Table 1). Subsequently, the compounds were tested on the healthy HDF cell line using 5 and 10 µM concentrations, aligning closely with their IC50 values. The MTT assay results, depicted in Supplementary Fig. S1, revealed that neither Au4MC nor Au6MC displayed cytotoxicity against HDF cells, as evidenced by the un-altered cell viability at the tested concentrations.

Furthermore, MCF7-treated samples were assessed for LDH activity at IC50 values. The results showed increased LDH activity on both Au4MC and Au6MC supernatants compared to the control group. DMSO concentrations of 0.5 %, which are known to cause MCF7 cell damage, raise LDH activity, were recorded as C+ (Fig. 3b).

### 3.3 Survival, hatching and cardiotoxicity on zebrafish embryos

**Fig. 4:**
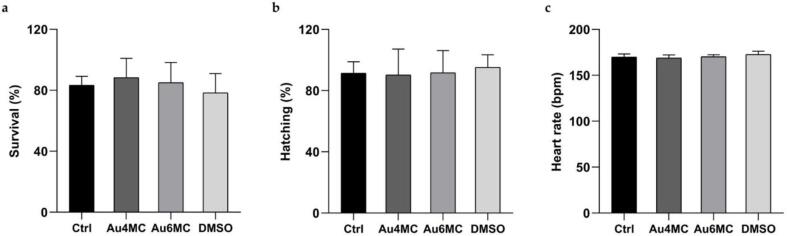
**Toxicity parameters**: (a) Survival (%) at 24 h of treatment; (b) Hatching (%) at 48 h of treatment; (c) Heart rate (bpm) at 72 h of treatment. No significant statistical difference was found (ANOVA followed by Tukey's test).

To evaluate the toxicity of Au4MC and Au6MC, zebrafish embryos were observed at 24 h, 48 h and 72 h of treatment. The results indicated that, at 24 h of treatment, none of the tested compounds caused any alterations in survival (Fig. 4a), which remained constant until 72 h of treatment. Similarly, the normal hatching process was also not affected (Fig. 4b); in fact, no early or delayed hatching occurred. Then, the heart rate measured at 72 h of treatment showed no alterations (Fig. 4c).

### 3.4 In vitro assessment of embryotoxicity on bovine embryo development

**Fig. 5:**
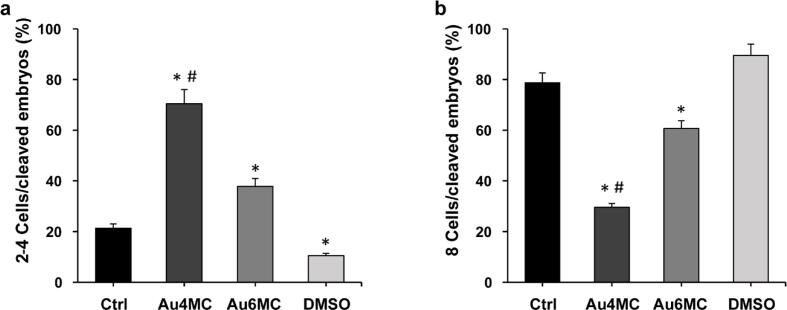
**Embryo stages after Au4MC and Au6MC treatments on d3 pi**. The graphs show the percentage of embryos at the 2–4 cell stage (a) and the 8-cell stage (b) on d3 p.i.. * p < 0.05 *vs* Ctrl, # p < 0.05 *vs* Au6MC.

**Fig. 6:**
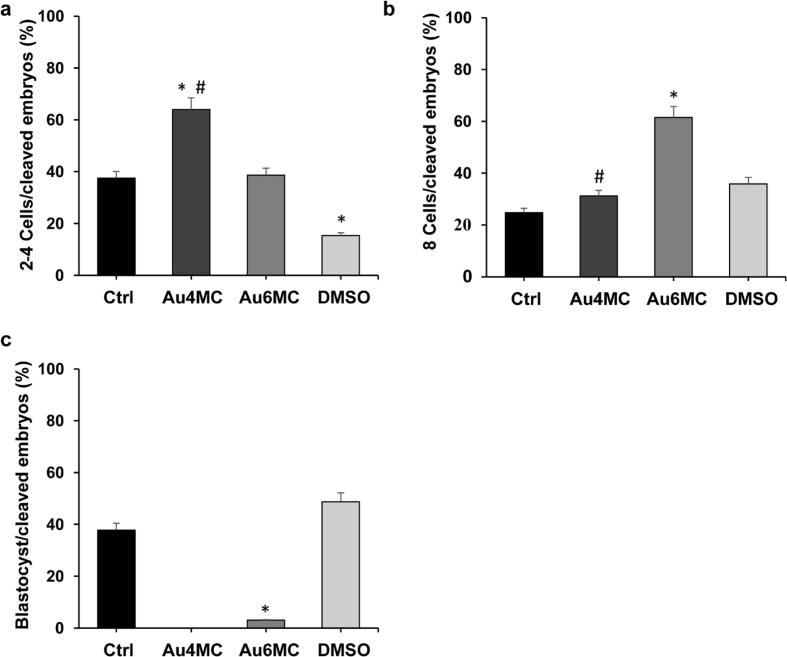
**Embryo stages after Au4MC and Au6MC treatments on d8 p**. The graphs depict the percentage of embryos at the 2–4 cell stage (a), the 8-cell stage (b) and blastocysts (c) on d8 p.i. * p < 0.05 *vs* Ctrl.

The in vitro fertilization experiments were designed to assess the impact of Au complexes (Au4MC and Au6MC) on bovine preimplantation development. Various developmental stages (2–4, 8 cells and blastocysts) were examined on d3 and d8 p.i. using stereomicroscopic observations. The fertilization rate was > 80 % for all tested samples (data not shown). Fig. 5a and b revealed that on d3 p.i., embryos treated with gold-based compounds (5.03 μM Au6MC or 5.53 μM Au4MC concentrations) exhibited a higher proportion of embryos at the 2–4 cell stage and a lower percentage of 8-cell stages compared to both the CTRL and DMSO groups. In particular, Au4MC displayed a significantly higher proportion of embryos at the 2–4 cell stage (70.4 %) and a lower percentage of 8-cell embryos (29 %) compared to the CTRL and DMSO groups, and Au6MC. Further, Au6MC exposure resulted in a slight increase in 2–4 cell stage (28 %) and a reduced 8-cell embryo rate (60 %) at d3 p.i.. On d8 p.i., Au4MC-treated samples exhibited a notably higher number of embryos at the 2–4 cell stage, suggesting a potential developmental block in the early stages (Fig. 6a and b). The number of 8-cell embryos was comparable to the controls, although blastocyst development did not occur (Fig. 6c). Au6MC showed a similar proportion of 2–4 cell embryos, a high percentage of 8-cell embryos and embryo development to the blastocyst stage, although significantly lower compared to the CTRL and DMSO samples. Moreover, the DMSO samples displayed embryonic development comparable to the control, indicating that the DMSO concentrations used for compound dissolution did not interfere with embryo development.

### 3.5 Evaluation of blastocyst developmental competency

**Fig. 7:**
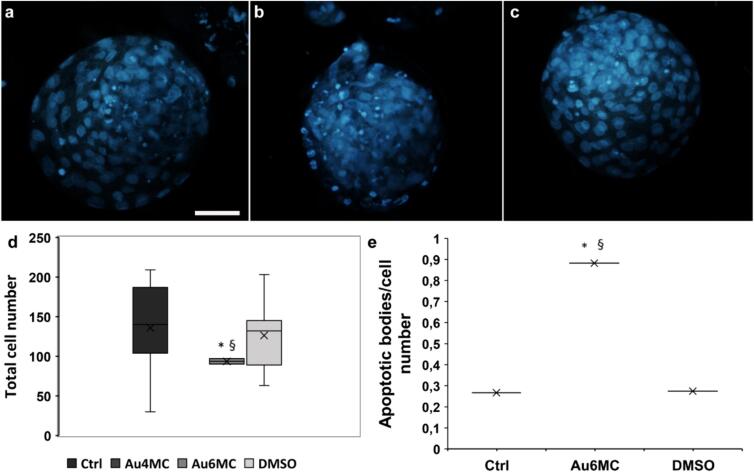
**Evaluation of Au compounds on bovine blastocysts competence** Representative micrographs of blastocysts on untreated sample (a, Ctrl), Au6MC-treated samples (b, Au6MC) and DMSO-treated sample (c, DMSO). d) The graph indicates the total cell number on blastocysts on un- and treated samples; e) the graph reports the apoptotic bodies to intact cell number ratio on blastocysts un- and treated with Au4MC or Au6MC. All the experiments were performed in duplicate, data are presented as means ± SD; * p < 0.05 *vs* Ctrl, § p < 0.05 *vs* C+.

To assess the competency and quality of blastocysts developed at d8 pi, the ratio of apoptotic bodies to intact cell nuclei and mean cell number on Hoechst-stained samples was evaluated on un- or treated (Au6MC) samples (Fig. 7). Blastocysts were not produced in the Au4MC-treated sample. Representative images of bovine blastocysts showed that the cell nuclei of non-treated embryos (Ctrl and DMSO) exhibited an oval shape with well-defined nuclei edges (Fig. 5a, c white arrow and d), whereas the apoptotic cells displayed chromatin condensation (pyknosis) and reduced nuclear sizes with bright fluorescence, ultimately leading to fragmentation into several small apoptotic bodies (Fig. 5a, c white arrowhead, and d). Blastocysts treated with Au6MC displayed a high number of apoptotic bodies (Fig. 7b, white arrowhead). As shown in the graph (Fig. 7d), the mean cell number of examined blastocysts ranged between 100 and 200 for both control and DMSO samples. However, the number of cells in blastocysts treated with Au6MC (93.5 ± 4.9) was significantly lower compared to both the control (146.4 ± 46.9) and DMSO (126.2 ± 40.2) samples.

### 3.6 Analysis of apoptosis on blocked embryos

**Fig. 8:**
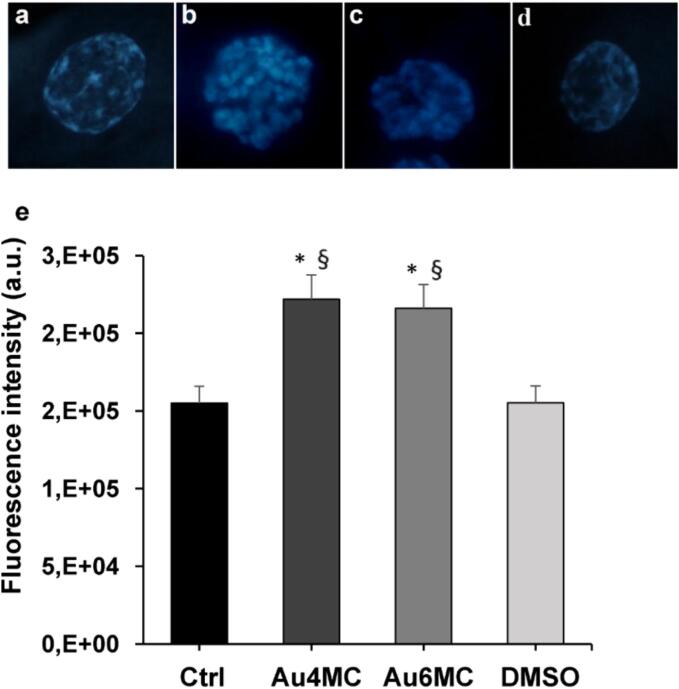
**Metabolic effect of Au compounds on bovine embryos** Representative images of blocked embryo’s nuclei on untreated sample (a, Crtl), Au4MC-treated samples (b, Au4MC), Au6MC-treated samples (c, Au6MC) and DMSO-treated sample (d, DMSO). e) The graph indicates the total fluorescence intensity of blocked embryo’s nuclei on un- and treated samples; All the experiments were performed in duplicate, data are presented as means ± SD; * p < 0.05 *vs* Ctrl, § p < 0.05 *vs* C + .

In order to assess apoptotic cells on blocked embryos, un- and treated samples were fixed and stained with Hoechst 33,342 at the end of the embryo culture and examined under a fluorescence microscope. Fig. 8a and d showed a representative healthy cell in blocked embryos with nuclei containing scattered small heterochromatic spots in CTRL and DMSO samples, respectively. On the other hand, blocked embryos treated with Au4MC or Au6MC displayed bright fluorescence of their cell nuclei, rich in heterochromatin (Fig. 8b and c). The total cell fluorescence intensity analysis revealed that the blocked Au4MC and Au6MC embryos had a greater total fluorescence intensity than the CTRL and DMSO groups (Fig. 8e). The proportion of apoptotic cells in blocked embryos did not change between the two chemicals.

## 4 Discussion

The identification of effective antitumor compounds certainly requires testing on various biological systems using sensitive and appropriate cytotoxicity assays. While studies on the cytotoxicity of newly synthesized drugs have predominantly conducted on somatic cell lines, limited research had addressed more sensitive biological systems, such as germ cell cultures, with a particular emphasis on embryonic development. As it is known, chemotherapeutic drugs act by disrupting certain cellular processes and altering the normal cell proliferation cycle, contributing to their detrimental impact on germ cells.

In our investigation, we studied two NHC-gold halide complexes derived from 4,5-diarylimidazoles, known for their good growth inhibitory effects against mammary and colon carcinoma cells (Liu et al., 2011b). Au4MC complex was previously tested on different cell lines, showing good cytotoxic ability although the thioredoxin reductases (TrxR) is not the main target like others gold complexes. Au6MC was designed in order to evaluate the steric hindrance on NHC ligands on the gold complex activity. These gold-based complexes were evaluated for their in vitro activity using a 2D culture model of cancer cells to confirm their selective activity. Once the most effective concentration of the compounds (IC50) was identified, their cytotoxic activity was assessed on both an *in vivo* zebrafish model and an in vitro bovine system using the in vitro fertilization technique (IVF). Bovine model shares notable similarities with human ovarian physiology and development, enabling the determination of cytotoxicity during the early stages of embryonic development.

Results revealed that Au4MC and Au6MC compounds, tested at the concentration of 5.53 μM and 5.03 μM, respectively, were proven to be non-toxic in zebrafish embryos. These results agreed with those obtained by Gao et al., (2017), regarding survival of embryos following exposure with AF but contrasted in terms of heart rate. Gao et al., (2017) found a decrease of heart rate from the concentration of 2.5 μM and a down regulation of oxidative stress genes. In fact, AF binds to TrxR, an enzyme involved in redox homeostasis. If the enzyme is not functioning, there is an increase in oxidative stress which results in decreased survival (Gao et al., 2017). Other studies where NHC-Au complexes have been tested, a mortality rate of almost 100 % was achieved at 100 μM and several phenotypic alterations were observed during zebrafish embryos development. One of these alterations was the decrease in the size of the yolk sac, resulting in nutrient shortages and, consequently, a decrease in the overall growth of the organism. The mechanism by which this occurs in zebrafish was unknown, but probably these complexes could interfere with cell proliferation (Farooq et al., 2015). So, in some cases these different compounds were proven to be harmless (Oehninger et al., 2013), in others toxic or even lethal (Farooq et al., 2015, Gao et al., 2017); these discordant results suggest that the toxicity of complex tested could depend on acetate group that could affect the overall toxic character (Farooq et al., 2015). The compounds tested in this work did not give the same toxic effects found in other work with similar compounds (Farooq et al., 2015, Gao et al., 2017). Recent studies have highlighted the susceptibility of pre-implantation embryonic development to various environmental factors, both *in vivo* (e.g., maternal nutrition) and in vitro (e.g., embryonic culture) (Fleming et al., 2004, Sinclair and Singh, 2007, Thompson et al., 2007, Watkins et al., 2008). Notably, toxicological studies on sensitive biological systems, such as germ cells and oocytes, have gained prominence (Rogers et al., 2021). For instance, Rogers et al., (2021) revealed the rapid degeneration of mouse oocytes and chromosomal abnormalities upon exposure to newly synthesized biomaterials (Rogers et al., 2021). Further studies emphasized the dose-dependent sensitivity of female gametes to external environmental factors, as demonstrated by meiotic abnormalities − particularly aneuploidies − induced by compounds like bisphenol A (BPA) in mouse oocytes (Hunt et al., 2003). Cattle oocyte culture studies revealed ovo- and embryo-toxic effects of Chlorpyrifos, impacting oocyte maturation, viability, and developmental competence (Nandi et al., 2011). However, there is limited literature available on the early stages of embryo development in *in vitro* models exposed to chemotherapeutic agents. Noteworthy studies on toxicological effects in sensitive biological systems, such as germ cells and oocytes, underscore the importance of understanding the potential risks associated with compounds during embryonic development. Examining the effects of chemotherapeutic agents (paclitaxel and cisplatin), Kim et al., (2019) observed significant reductions in follicle expansion and genetic downregulation related to mouse oocyte development (Kim et al., 2019). Similarly, Yang et al., (2021) investigated the toxic effects of Cyclophosphamide (CTX) on mouse follicular and oocyte development, revealing reduced primordial follicles, meiotic abnormalities, increased reactive oxygen species (ROS) levels, and impaired embryonic development in terms of cleavage and blastocyst percentages highlighting the adverse impact of drug exposure on embryonic development (Yang et al., 2021). Furthermore, there is a well-established correlation between rising oxidative stress levels and a decrease in embryo cleavage rates (Agarwal et al., 2022). Early cleavage and the developmental progression to the 8-cell stage are crucial for successful outcomes. Previous studies in cattle have reported paternal influence on both developmental embryo kinetics and molecular profile (Ward et al., 2001, Guibu de Almeida et al., 2022). In our study, we observed notable differences in the 2–4 and 8-cell stage embryos treated with Au compounds, suggesting a potential delay or arrest in cleavage, potentially induced by paternal DNA repair in the zygote. Our findings specifically highlighted that the Au4MC compound induces an early blockage in embryonic development at + 3 days, characterized by a higher percentage of embryos at 2–4 cells compared to 8 cells. On the other hand, Au6MC displayed a slightly more advanced blockage, exhibiting a higher percentage of 8-cell embryos. Further analyses conducted on Au4MC at the end of embryonic culture confirmed a developmental arrest specifically at the 2–4 cell stage, with no production of blastocysts. Interestingly, despite its higher percentages of 8-cell embryos, Au6MC displayed a significantly reduced percentage of blastocysts, characterized by a lower cell count compared to the control group. This finding strongly suggests a reduced blastocysts competence (Loskutoff et al., 1993) associated with Au6MC treatment. Overall, these results indicated that early cleaving human and bovine zygotes have a higher likelihood of advancing to the blastocyst stage, indicating that the administration of Au compounds does not support embryo development.

Another aspect to take in account is the blastocyst quality that profoundly affects the potential for implantation and successful development to term. Among mammalian species, the blastocyst quality is characterized by the highest occurrence of spontaneous apoptotic processes, which play a crucial protective role by removing genetically abnormal blastomeres (Anifandis et al., 2015). In the context of bovine reproduction, in vitro-produced blastocysts generally exhibit a higher rate of TUNEL-positive cells compared to their *in vivo* counterparts (Osman et al., 2015). Our research outcomes unequivocally highlighted that the treatment of zygotes with Au6MC resulted in a substantial increase in apoptosis within the blastocyst. This finding underscores the noteworthy impact of chemotherapeutic agents on blastocyst DNA fragmentation, aligning with existing literature (Wang et al., 2012). Furthermore, since we observed that the blockage of embryos occurred in the initial developmental phases, we evaluated the degree of chromatin compaction in embryos blocked at 2–4 cells and 8 cells. We found a notable increase in pyknotic nuclei full of heterochromatin in blocked embryos compared to the control groups. We hypothesize that this apoptosis is secondary to oxidative stress, given its potential role in repairing damage resulting from an insult (Liang et al., 2013).

This study seeks to provide valuable insights into the embryonic toxicity of gold-based compounds and their effects during early developmental stages. Understanding the potential risks associated with these compounds during embryonic development is crucial for their safe use in chemotherapy. The outcomes of this research will contribute to the field of toxicology and assist in the development of new cytotoxicity tests.

## 5 Conclusion

In conclusion, our study goes beyond delineating the selectivity and cytotoxicity of gold-based compounds on tumor cells, extending to unveil their varied impacts on zebrafish and bovine embryos. This comprehensive exploration not only deepens our understanding of embryonic toxicity but also carries significant implications for the secure application of these compounds in chemotherapy. The findings serve to provide a crucial foundation for advancing research in toxicology and developing innovative cytotoxicity tests. This, in turn, ensures a more nuanced comprehension of the intricate effects of these compounds during early embryonic development, paving the way for enhanced safety assessments and informed clinical applications.

## Declaration of competing interest

The authors declare that they have no known competing financial interests or personal relationships that could have appeared to influence the work reported in this paper.

## Data Availability

Data will be made available on request.
