# Supplementary material for: Insight on cytotoxic NHC gold(I) halide complexes evaluated in multifaceted culture systems
Source: Curr Res Toxicol. 2024 May 23;6:100174. doi: 10.1016/j.crtox.2024.100174 (PMC11152893; doi:10.1016/j.crtox.2024.100174)
Supplement: Supplementary Data 1 [file mmc1.docx]

**Insight on Cytotoxic NHC Gold(I) halide complexes evaluated in multifaceted culture systems**

Vincenza De Gregorio^1§^, Alessandra La Pietra^1§^, Andrea Candela^1^, Carlo Oliviero^2^, Ida Ferrandino^*†1^, Diego Tesauro^*†3^

^1^ Department of Biology University of Naples “Federico II”, via Cinthia 80126, Napoli, Italy

^2^ Department of Experimental Medicine, Section of Biotechnology, Medical Histology and Molecular Biology, University of Campania "Luigi Vanvitelli", 80138 Naples, Italy

^3^Department of Pharmacy and Interuniversity Research Centre on Bioactive Peptides (CIRPeB), University of Naples “Federico II”, via Montesano 49, 80131 Naples, Italy.

^*^Correspondence: ferrandi@unina.it (I.F.); dtesauro@unina.it (D.T.)

^§^ These authors contributed equally to this work.

^†^These authors contributed equally to this work and share senior authorship.

**Supplementary results**

**Figure S1**


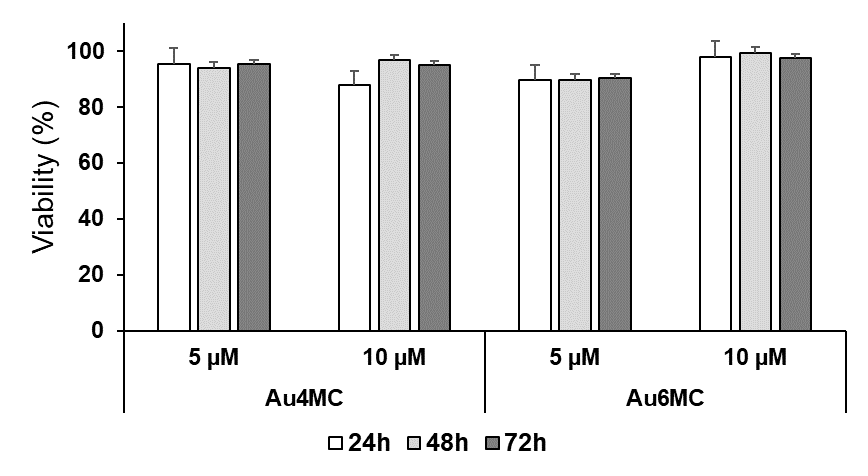


**S1. Effects of Au4MC or Au6MC treatment on cell viability on HDF cell line.**

HDF cells were treated with different concentrations of Au4MC or Au6MC for 24, 48, and 72 h. Cell viability was determined by using an MTT assay. Data are expressed as the percentage of control cells and are the means ± SD of two independent experiments, each performed in triplicate.

**Supplementary figure S1**

In order to assess cell viability the compounds were tested on the healthy HDF cell line using 5 and 10 µM concentrations, aligning closely with their IC50 values. The MTT assay showed that neither Au4MC nor Au6MC displayed cytotoxicity against HDF cells, as evidenced by the unaltered cell viability at the tested concentrations.

**Figure S2**


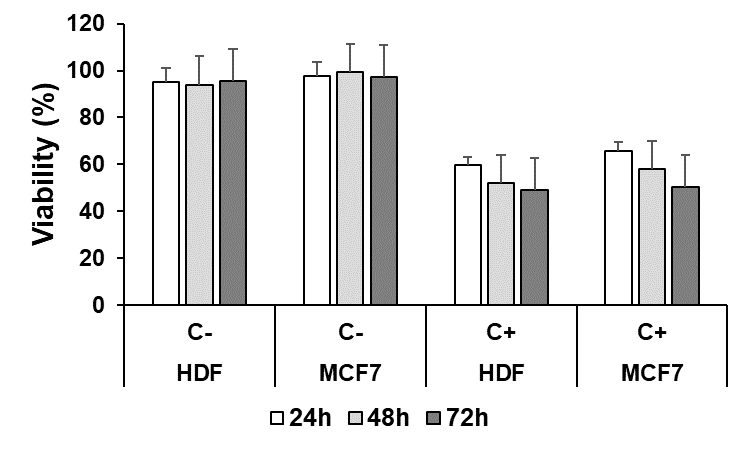


**S2. Effects of DMSO on healthy (HDF) and diseased (MCF7) cell lines.**

The graph represents the negative (C-, untreated cells) and positive (C+, DMSO 50%) controls at 24, 48 and 72 hrs after incubation. MTT test was used to assess cell viability. Data are expressed as the percentage of control cells and are the means ± SD of two independent experiments, each performed in triplicate.

**Supplementary Figure S2**

Cell viability was assayed on both healthy (HDF) and diseased (MCF7) cell lines using 50% DMSO as C+ determining a reduction of cell viability in comparison to the control samples (C-, untreated sample) at different time points.
